# Reduced angiovasculogenic and increased inflammatory profiles of cord blood cells in severe but not mild preeclampsia

**DOI:** 10.1038/s41598-021-83146-8

**Published:** 2021-02-11

**Authors:** Seonggeon Cho, Young-Doug Sohn, Sangsung Kim, Augustine Rajakumar, Martina L. Badell, Neil Sidell, Young-sup Yoon

**Affiliations:** 1 Coulter Department of Biomedical Engineering, Emory University and Georgia Institute of Technology, Atlanta, GA 30322 USA; 2 Division of Cardiology, Department of Medicine, Emory University School of Medicine, 101 Woodruff Circle, Woodruff Memorial Building (WMB) 3309, Atlanta, GA 30322 USA; 3 Department of Gynecology and Obstetrics, Emory University, Atlanta, GA USA; 4 Severance Biomedical Science Institute, Yonsei University College of Medicine, Seoul, South Korea

**Keywords:** Pre-eclampsia, Angiogenesis, Prognostic markers

## Abstract

Preeclampsia (PE) is a prevalent pregnancy disorder that leads to high maternal and fetal morbidity and mortality. While defective vascular development and angiogenesis in placenta are known as crucial pathological findings, its pathophysiological mechanism remains elusive. To better understand the effects of PE on angio-vasculogenesis and inflammatory networks in the fetus and to identify their biological signatures, we investigated the quantitative and functional characteristics of cord blood-derived mononuclear cells (CB-MNCs) and CD31-positive MNCs. Flow cytometry analysis demonstrated that the CB-MNCs from the severe PE group had significantly decreased number of cells expressing CD3, CD11b, CD14, CD19, KDR, and CD31 compared with the normal group. Quantitative real time PCR (qRT-PCR) shows down-regulation of the major angiogenic factor *VEGFA* in MNCs and CD31^+^ MNCs in severe PE*.* The major inflammatory cytokines IL1 was highly upregulated in CD31^+^ CB-MNCs in the severe PE patients. Mild PE patients, however, did not display any significant difference in expression of all measured angiogenic genes and most inflammatory genes. These findings show distinct angiogenic and inflammatory signatures from severe PE, and they may play a significant role in the pathogenesis of vascular defects in placenta of severe PE.

## Introduction

Preeclampsia (PE) is a pregnancy-related clinical syndrome that affects 3–5% of all pregnancies^1^ and is a leading cause of maternal and perinatal morbidity and mortality worldwide^2^. The onset of the disease varies from 20 weeks of gestation up to 6 weeks after delivery (postpartum preeclampsia)^3^, but the severity increases for the patients with gestational age less than 34 weeks. This multisystem disorder is characterized by high blood pressure as a consequence of endothelial cell dysfunction. The dysfunctional vessels prevent appropriate flow exchange and induce oxidative stress damaging key organs such as brain, heart, kidneys, liver, and eyes. Pregnant women with early-onset severe PE are of particular concern since they are more likely to bear a baby with intrauterine growth retardation (IUGR)^4^. Defective deep placentation due to absent or inadequate physiological transformation of myometrial segment of the spiral artery limits placenta blood flow and restricts maternal and fetal nutrition exchange^5^. Fetuses with IUGR from preeclamptic women have elevated risk of stillbirth^6,7^, and even after their successful delivery, IUGR infants have high incident rates of metabolic syndrome and ischemic heart diseases in adulthood^8,9^. Despite its long history of public health significance and intense study, the pathogenesis of PE is still poorly understood, and therapeutic options are limited to delivery to prevent further maternal or fetal complications from disease progression. ^10^.

Recent studies have demonstrated that maternal–fetal genotype incompatibility, uncontrolled hypoxia, and immune dysregulation contribute to abnormal decidual and trophoblast interaction and shallow placentation^11^. The placental dysfunction is complicated with oxidative and endoplasmic reticulum stress, which causes trophoblast apoptosis and release of microparticles and nanoparticles. Elevated number of reactive oxygen species and proinflammatory cytokines in the maternal circulation escalate the leukocyte accumulation in the blood vessel and the chance of convulsion. Under the influence of such circumstances, endothelial cells malfunction and angiogenesis becomes defective^11^.

Major studies have attempted to demonstrate the diagnosis and prognosis of PE with biomarkers found from maternal blood circulation related to vascular dysfunction and angiogenic defects. Levels of antiangiogenic factors including soluble vascular endothelial growth factor receptor 1 (sVEGF-1 or sFLT1) and soluble endoglin (sEng) are significantly increased in the serum of PE patients with subsequent decreases of vascular endothelial growth factor A (VEGFA) and placental growth factor (PGF)^12,13^. Some studies reported that endothelial progenitor cell (EPC) from maternal circulation decreased in PE conditions ^14,15^. Circulating CD34^+^KDR^+^ and CD133^+^KDR^+^ EPC were significantly lower in women with PE^14^. It is also reported that circulating EPC from PE patients is functionally deficient, having a lower number of endothelial colony-forming units (CFU)^15,16^. However, little is known about the effects of PE on hematopoietic and vascular cells derived from the developing fetus. It is reported that fetal hematopoiesis is affected as a consequence of PE itself rather than growth restriction alone^17–19^. The number of hematopoietic stem cells is reduced in preeclamptic cord blood (CB) with a concomitant reduction of endothelial colony-forming cells^20^.

A major goal of recent investigations has been to identify CB cells from women with PE that are underrepresented or abnormally functioning and that could account for the inadequate growth of blood vessels in and from PE placentas^11^. Researchers have anticipated that such cells and their gene expression profiles may serve as biomarkers to predict the presence and/or severity of PE. Previously, we demonstrated that CD31^+^ mononuclear cells (MNCs) derived from human peripheral blood represent a broad spectrum of circulating cells which possess angiogenic and vasculogenic properties^21,22^. Further studies demonstrated that the number and angiogenic properties of CD31^+^ cells are reduced in patients with coronary artery disease^23^. Accordingly, we have postulated that the impaired vascular development and angiogenic activities of the placenta in PE may be closely associated with the number and function of CD31^+^ cells and/or other subpopulations of CB-MNCs which are involved in angio-vasculogenic activities. Therefore, we sought to identify abnormalities in subpopulations of CB-MNCS from PE patients and further to investigate whether there are any significant alterations in the expression of genes that may, at least partially, account for abnormal blood vessel formation and inflammation associated with PE placenta.

## Results

### Maternal and fetal conditions of severe preeclampsia

Based on the American College of Obstetricians and Gynecologists (ACOG) guidelines and the patients’ diagnostic results obtained from the department of Obstetrics and Gynecology of Emory University, we divided the PE patients into two conditions, mild and severe PE (Table 1). The patients’ systolic blood pressure (BP) matched the range defined from ACOG guidelines based on their disease severity^24^. All the patients with severe PE experienced early-onset of the disease, and therefore had early delivery due to their acute symptoms. Excess plasma sFLT1 were found from both conditions, but there was not a clear distinction found in their levels. Severe PE had significantly lower placental weight (*P* = 0.0008) and birth weight (*P* < 0.0001) compared to both normal pregnancy and mild PE, and such conditions support the particular concern that the pregnant women with early-onset severe PE are more likely to bear a baby with IUGR^25^.

**Table 1:** Characteristics of the study population at delivery.

Variables	Normal (n = 12)	Mild PE (n = 5)	Severe PE (n = 6)
Systolic BP (mmHg)	115 ± 8.89	152 ± 16.6***	170 ± 16.8***
Diastolic BP (mmHg)	71 ± 9.7	94 ± 4.1**	94 ± 14***
Maternal plasma sFlt1 (pg/mL)	5737 ± 4032	26,310 ± 17,900*	30,450 ± 17,380**
Placental Weight (g)	652 ± 120	661 ± 222	317 ± 141***^/##^
Birth Weight (g)	3366 ± 315	3139 ± 857.6	1597 ± 813.8***^/##^
Maternal age (years)	34 ± 6.0	34 ± 3.4	31 ± 4.3
Maternal pre-pregnancy BMI (kg/m^2^)	33.41 ± 11.55	32.02 ± 3.698	30.97 ± 4.691
Smoking	0	0	0
Gestational delivery week	39.1 ± 1.08	37.3 ± 2.19	32.1 ± 3.52***^/##^
Newborn sex (F/M)	4/8	2/3	5/1
Proteinuria	NA	100%	100%
Mode of delivery (C-section/Vaginal)	9/3	3/3	5/1
Induction of labor (yes/no)	2/7	4/1	2/3

Data are presented as mean ± SEM. Patient variables were analyzed by one-way ANOVA followed by Tukey’s multiple comparison. *P < 0.05, **P < 0.01, ***P < 0.001 for mild PE versus normal or severe PE versus normal; ^#^*P* < 0.05, ^##^*P* < 0.01, ^###^*P* < 0.001 for severe PE versus mild PE. Proteinuria measurement was based on a combination of 24 h urine test and protein/creatinine ratio. *BP* blood pressure, *BMI* body mass index, *NA* not applicable.

### Reduced subpopulations of CB-MNC in severe PE

**Figure 1:**
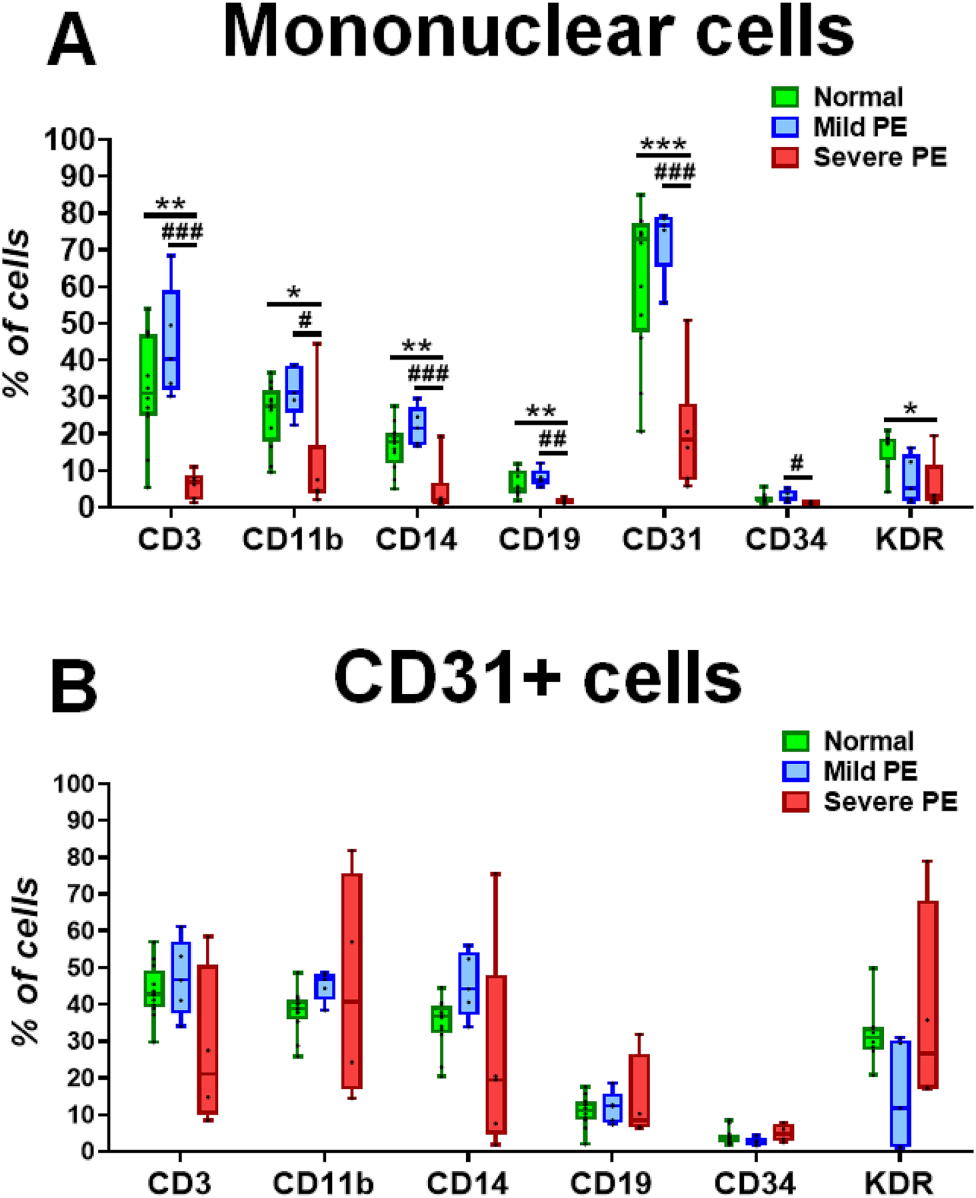
Difference in subpopulations of whole cord blood mononuclear cells and MACS-sorted CD31^+^ cells between normal, mild PE, and severe PE groups. Flow cytometry analysis for the indicated surface markers. (**A**) Flow cytometry analysis performed on CB-MNCs from normal (n = 8–12), mild PE (n = 5), and severe PE (n = 5–6) groups. (**B**) Flow cytometry analysis performed on CB-CD31^+^ cells from normal (n = 8–12), mild PE (n = 5), and severe PE (n = 4–5) groups. Statistical analyses were performed with one-way ANOVA followed by Tukey’s multiple comparison. *P < 0.05, **P < 0.01, ***P < 0.001 for severe PE versus normal; ^#^*P* < 0.05, ^##^*P* < 0.01, ^###^*P* < 0.001 for severe PE versus mild PE; ^$^*P* < 0.05, ^$$^*P* < 0.01, ^$$$^*P* < 0.001 for mild PE versus normal.

We performed flow cytometry analysis to determine whether any subpopulations within MNCs are changed in PE patients compared to the normal counterpart. We included surface markers such as CD3, CD11b, CD14, CD15, CD31, CD34, and KDR in this analysis (Fig. 1). Particularly, CD31 was included as a marker to indicate an angio-vasculogenic subpopulation for this study. In the MNC population, compared to both mild PE and normal groups, the severe PE group had significantly lower number of cells expressing CD3, CD11b, CD14, CD19, KDR and CD31 (*P* < 0.05 for all) (Fig. 1A). There was no significant difference between normal pregnant and mild PE groups in the number of these subpopulations. These data indicate that most subpopulations of the MNCs are decreased in severe PE only. Next, we determined the percentage of cells expressing the above markers among CD31^+^ cells. Again, between normal and mild PE, there was no significant difference (Fig. 1B). Within the CD31^+^ population, CD14^+^ cells, which are known to give rise to EPCs^26^ were decreased in severe PE compared to the normal and mild PE while it barely missed the statistical significance (*P* = 0.067).

### Reduced expression of angiogenic factors and increased expression of inflammatory genes in cord blood MNCs and CD31^+^ cells

**Figure 2:**
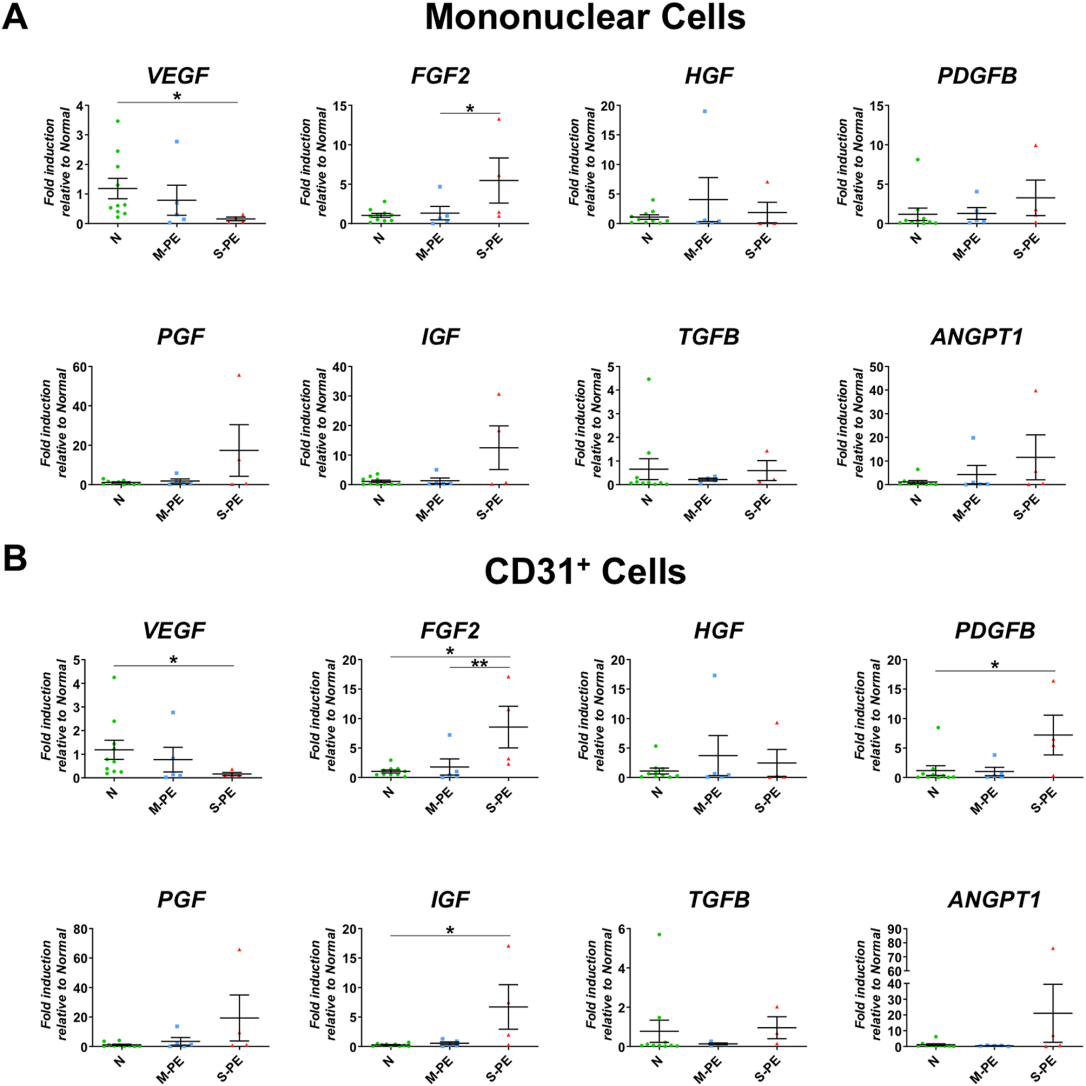
Differential angiogenic gene expression of cord blood mononuclear cells and CD31^+^ cells between normal, mild PE and severe PE groups. (**A**) qRT-PCR analysis performed on CB-MNCs from normal (n = 10), mild PE (n = 5), and severe PE (n = 4) groups. (**B**) qRT-PCR analysis performed on CB-CD31^+^ cells from normal (n = 10), mild PE (n = 5), and severe PE (n = 4) groups. Each dot in the plots is the triplicated sample data analyzed by qRT-PCR. Statistical analyses were performed with Kruskal–Wallis test followed by uncorrected Dunn test for pairwise comparison. *P < 0.05, **P < 0.01, ***P < 0.001.

We then compared the expression patterns of angiogenic genes and inflammatory genes in CB-MNCs and CB-CD31^+^ cells between the normal, mild PE, and severe PE groups by qRT-PCR. We first measured angiogenic gene expression such as VEGFA, FGF2, CXCL12, PDGFB, PGF, IGF, TGFB, and ANGPT1. In the CB-MNC, between the normal and mild PE, there was no significant difference in gene expression; however, the severe PE displayed significance differences in gene expression levels compared with both the normal and mild PE (Fig. 2A). In the severe PE, the mRNA expression of the major angiogenic factor *VEGFA* was substantially reduced. We next examined expression levels of the same genes in CD31^+^ cells. Normal and mild PE did not show any significant difference in the angiogenic gene expression profile. Severe PE demonstrated significantly reduced expression of *VEGFA* and increased expression of other angiogenic factors such as *FGF2*,* PDGFB*, and* IGF* compared to the normal group. In general, difference in angiogenic gene expression was more significant in CB-CD31^+^ cells than those observed in CB-MNCs, suggesting the importance of CD31^+^ cells in reflecting the changes of angiogenic factors in severe PE (Fig. 2B).

**Figure 3:**
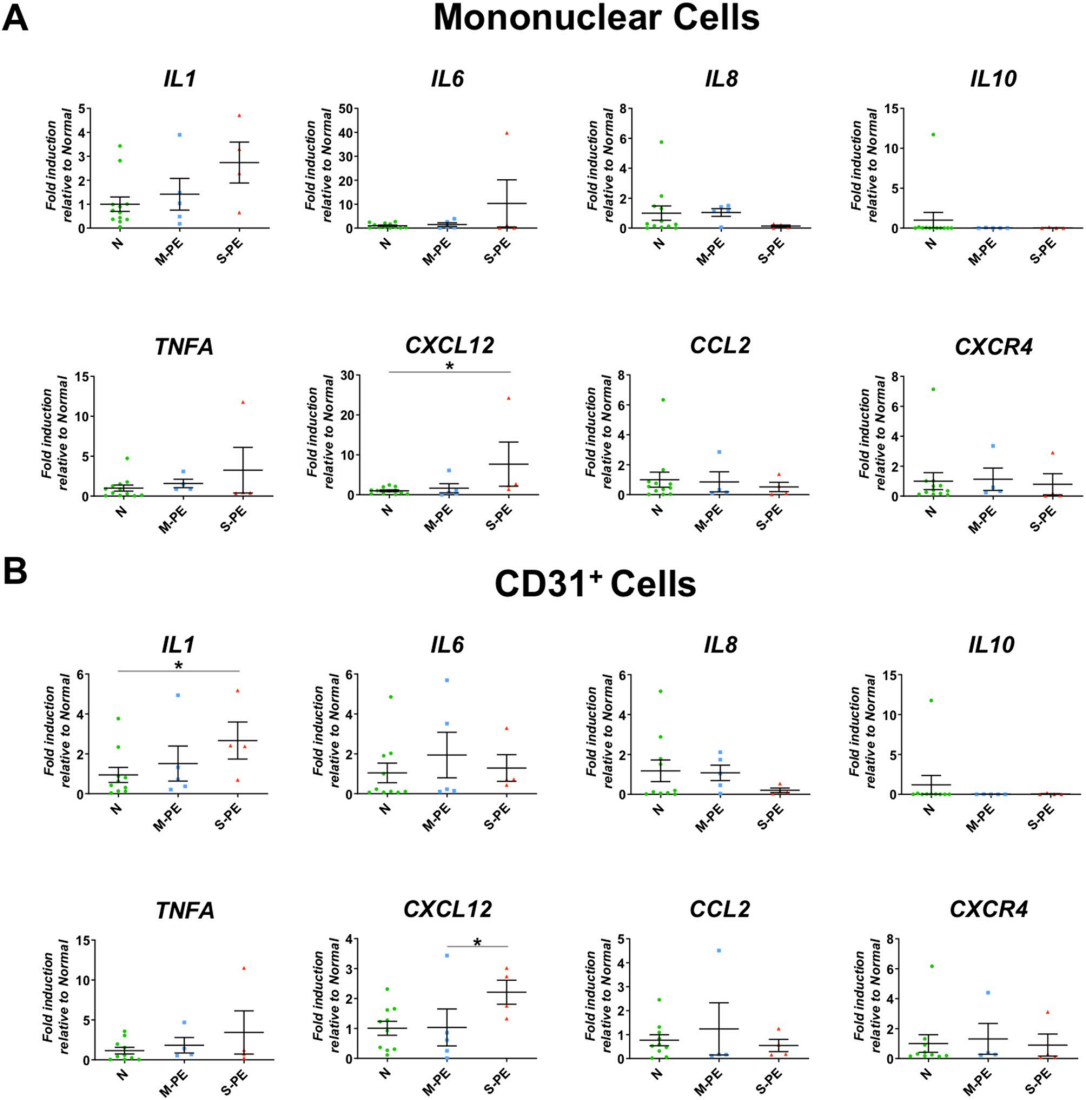
Differential inflammatory gene expression of cord blood mononuclear cells and CD31^+^ cells between normal, mild PE and severe PE groups. (**A**) qRT-PCR analysis performed on CB-MNCs from normal (n = 10), mild PE (n = 5), and severe PE (n = 4) groups. (**B**) qRT-PCR analysis performed on CB-CD31^+^ cells from normal (n = 10), mild PE (n = 5), and severe PE (n = 4) groups. Each dot in the plots is the triplicated sample data analyzed from qRT-PCR. Statistical analyses were performed with Kruskal–Wallis test followed by uncorrected Dunn test for pairwise comparison. *P < 0.05, **P < 0.01.

We next investigated the mRNA expression of pro-inflammatory genes IL1, IL6, IL8, IL10, TNFA, CCL2, CXCR4, and CXCL12. In MNCs, *CXCL12* expression was significantly elevated and *IL1* and *IL6* expression showed an increased trend in severe PE compared to the normal (Fig. 3A). In CD31^+^ cells, *IL1 and CXCL12* expression was higher in severe PE compared to the normal while statistical significance was found only in *IL1* (Fig. 3B). Together, these data indicate that severe PE is associated with reduced expression of VEGFA, a main angiogenic factor and increased expression of major inflammatory factors *IL1* and *CXCL12*.

## Discussion

This study demonstrated that in severe PE, but not mild PE, there is a decrease in various subpopulations of CB-MNCs including CD31^+^ cells along with a reduction in VEGF-A gene expression and increased inflammatory gene expression. These results are consistent with the fact that while placental vascular development is relatively preserved in mild PE, premature or shallow placentation is presented as a common pathological feature of severe PE. Severe PE patients are often associated with IUGR. Indeed, half of the patients with severe PE bore children with IUGR while one fifth for mild PE and none for normal pregnancy^27^. Studies reported that early-onset/severe PE has defective fetal vasculature and this may contribute to IUGR^28,29^. Fetuses from early-onset PE with IUGR had defective cardiovascular function, and they presented a higher chance of having ischemic cardiovascular disease post-delivery^1,30^. Since fetal vascular development depends on the adequate supply of angiogenic factors, a reduced number and function of CB-derived angio-vasculogenic cells could underlie impaired vascular development or angiogenesis in fetus and placenta of severe PE^31^. As such, the CD31^+^ cell fraction and its gene expression profile reflect the vascular pathophysiology of pre-eclampsia and may serve as a biomarker for severe PE.

Impaired fetal hematopoiesis has been frequently reported from preeclamptic patients. Reduced number of EPCs under such circumstances is an inevitable consequence. However, due to rarity and variability in culture, the use of EPCs as biomarkers for vascular diseases has been controversial and has not been widely used in clinical settings^32–35^. Alternatively, CD31^+^ cells have emerged as a biomarker representing an angio-vasculogenic population among MNCs due to its broad role in vessel formation^23^. Our data demonstrated that CD31^+^ cells, CD14^+^ cells, CD11b^+^ cells^36^, and CD3^+^ cells^37^ among others were significantly reduced in CB-MNCs of severe PE patients, among which CD31^+^ cells and CD3^+^ cells showed the most notable changes. CD31^+^ cells are a collection of angio-vasculogenic effector cells, as they include various populations of cells related to vessel formation and regeneration including EPCs^21,22,38^. Moreover, CD14^+^ cells are decreased in the MNCs and the CD31^+^ cell fraction from severe PE patients (Fig. 1B). In previous cardiovascular studies, CD14^+^ MNCs were shown to yield cells with an endothelial characteristic that has a functional role in neovascularization^39^. Thus a reduced number of CD14^+^ cells in cord blood further suggest the impaired angio-vasculogenic function of CB-MNCs that is closely associated with severe PE.

Further supporting our hypothesis of the important angiogenic role of CD31^+^ cells in placental development is the lower expression of the crucial angiogenic factor VEGFA in both CB-MNCs and CB-CD31^+^ cells from severe PE patients (Fig. 2). Previous studies have shown that a CD31^+^ population in peripheral blood MNCs are a main source of angiogenic factors such as VEGFA^21,22,38^. We have now documented that CB-MNCs and CB-CD31^+^ cells from PE patients show markedly reduced expression of *VEGFA* consistent with the defects in vascular development of PE placenta. These results correspond well with prior studies demonstrating elevation of sFLT-1/sVEGF-1 in the maternal circulation and placenta, which are inhibitors of VEGF signaling^12,13^ That the other angiogenic factors analyzed showed increased expression in PE is interesting and may be a compensatory effects resulting from the decreased expression of VEGFA and angiogenesis in PE placenta. Together this imbalance could underlie defective vessel formation in placenta. We also found that the crucial inflammatory cytokine IL-1 and CXCL12 were increased in CB-MNCs and CB-CD31^+^ cells (Fig. 3).

PE is characterized by chronic inflammation, oxidative stress, and autoantibodies^40,41^. Previous reports have demonstrated reductions in angiogenic genes and disturbance in inflammatory gene regulation in the placenta of severe PE^9^. Our data obtained from CB cells further support this pathophysiology of defective placental vascular development and suggest that CB-CD31^+^ cells and their gene expression patterns have pathophysiological significance that can potentially serve as a biomarker for severe PE. Following these clues in future studies of gene expression profiles of CB cells at the whole genome level may provide valuable insight to help understand the role of perturbed cellular profiles and abnormally expressed genes in the etiology of severe PE in order to develop novel markers and therapeutic modalities.

There are several limitations in our study. One of the caveats is that we address changes in markers in cord blood post-delivery. Utero-placental development is established in first trimester of pregnancy. Thus, the contribution of these defects in early placental development could not be addressed. However, previous studies indicated that patients with early-onset preeclampsia present signs of utero-placental ischemia as early as first trimester of pregnancy^42,43^. In addition to placental vascular deficiency, abnormal umbilical cord vessels were reported for patients with severe PE^28,29^. It is not coincidental that early-onset/severe preeclamptic CB has reduced number and function of angio-vasculogenic cells that play an essential role in vascular development. Later studies can be designed to address this question by collecting early pregnancy samples and characterize them for changes in cell numbers and marker genes. We also recognize the limited number of the severe PE samples. The current sample size of severe PE was reached without deliberate sample selection. PE affects 3–5% of all pregnancy, and 0.3% of pregnant women develop severe PE^1,44^. The small sample size of severe PE was inevitable with the random sampling.

## Perspectives

Impaired fetal hematopoiesis has been reported from preeclamptic patients. However, the underlying mechanisms are unclear. This study analyzes subpopulations of cord blood-derived mononuclear cells and CD31^+^ cells and elucidates the effects of preeclampsia on angio-vasculogenic and inflammatory properties of the cord blood. Our study demonstrated that severe PE, but not mild PE, is associated with a decrease in various subpopulations of CB-MNCs including CD31^+^ cells along with changes in angiogenic and inflammatory gene expression. The reduced number and function of CB-derived angio-vasculogenic cells could underlie impaired vascular development or angiogenesis in placenta and fetus of severe PE, which could result in impaired fetal development. These results further imply that the CD31^+^ cell fraction and its gene expression profile could reflect the unique pathophysiology of severe PE, which is different from mild PE.

## Materials and methods

### Inclusion of preeclampsia patients

CB of healthy volunteers and PE patients were obtained from Department of Obstetrics and Gynecology of Emory. A total of 23 samples consisting of 12 normal and 11 PE samples were included (Table 1). Emory Institutional Review Board (IRB00078902) approved the study protocol and patients provided informed consent. All experiments were performed in accordance with relevant guidelines and regulations. Patients were approached for enrollment in the study at the time of preeclampsia diagnosis and were not deliberately selected based on the severity of PE or the gestational age. When we had reached the current sample size, the patients were categorized based on the severity of diagnosis and analyzed accordingly. PE was defined according to the 2017 ACOG Hypertension Guidelines^24^. Amongst the diseased samples, we further dichotomized mild preeclampsia (systolic blood pressure ≥ 140 mmHg and/or diastolic blood pressure ≥ 90 mmHg with late-onset disease development > 34 weeks) and severe preeclampsia (systolic blood pressure ≥ 160 mmHg and/or diastolic blood pressure ≥ 110 mmHg with early-onset disease development < 34 weeks) based on the standard diagnostic criterion provided from the ACOG guidelines. Five of them were identified as severe PE and six of them were identified as mild PE. Our inclusion criteria include non-smoking, English-speaking women older than 18 years. Patients with preexisting chronic conditions (such as heart disease, diabetes, pre-gestational hypertension, autoimmune disorders), pregnancy complications besides PE, or any known fetal anomalies were excluded. Normotensive pregnancies served as controls.

### Cord blood mononuclear cell isolation and magnetic-activated cell sorting

Cord blood was collected immediately after delivery of the placenta using a sterile collection unit (PALL Medical, Pall International Sarl, Fribourg, Switzerland). Each bag contains 35 ml of anticoagulant citrate phosphate dextrose solution and can collect 210 ml of cord blood. The bags were transported to the research facility within an hour for processing. From the cord blood, we isolated MNCs as previously reported with some modifications^21,45^. CB was centrifuged at 1340*g* for 25 min to obtain cell pellets, which then were suspended in autoMACS Rinsing Solution (PBS with 0.5% BSA and 2 mM EDTA, pH 7.2) (Miltenyi Biotec, Auburn, CA, USA). The diluted cell suspension was layered over 5 ml of Histopaque-1077 (Sigma, St. Louis, Missouri) and centrifuged at 400*g* for 30 min. The MNCs were harvested from the interface and washed with autoMACS buffer. The washed cells were counted and resuspended in autoMACS buffer, FcR blocking reagent and human CD31 microbeads (Miltenyi Biotec, Auburn, California) were added, and cells were incubated at 4 °C for 15 min. The cells were again washed and resuspended in MACS buffer. Following the manufacturer’s protocol, CD31^+^ and CD31^-^ cells were separated by auto MACS Separator (Miltenyi Biotec, Auburn, CA, USA) using a POSSEL program. MACS-sorted cells were sampled separately for the later experiments for flow cytometry and quantitative real-time RT-PCR.

### Flow cytometry

Flow cytometry was performed as described previously^46^. Briefly, cells were resuspended in PBS and incubated for 30 min at 4 °C with antibodies as indicated below. After staining, the cells were analyzed on a flow cytometer (BD FACS Canto II, BD Biosciences, CA, USA). The following antibodies were used: PECAM1-PE (130-092-653, Miltenyi Biotec, CA, USA), CD14-PE (555398, BD Biosciences, CA, USA), CD11b-PE (555388, BD Biosciences, CA, USA), CD34-PE (555822, BD Biosciences, CA, USA), KDR-PE (FAB357P, R&D Systems, MN, USA), CD3-FITC (555916, BD Biosciences, CA, USA), CD4-FITC (555346, BD Biosciences, CA, USA), CD19-FITC (555412, BD Biosciences, CA, USA), CD15-FITC (555401, BD Biosciences, CA, USA) and appropriate isotype control antibodies. Flow cytometric data were analyzed with FlowJo (Tree Star, Inc., Ashland, OR, USA) using appropriate isotype-matched controls.

### Quantitative real time RT-PCR

Total RNA was isolated with the RNeasy Plus Mini Kit (Qiagen) according to the manufacturer’s instructions. Based on the varying volumes of the cord blood, the number of cells and the amount of isolated RNAs were different. Thus, we used 100 ng to 1 mg RNA based on availability. The extracted RNA was reverse transcribed into cDNA via Taqman reverse transcription reagents, including random hexamers, oligo (dT), and MultiScribe MuLV reverse transcriptase (Applied Biosystems). Quantitative real-time RT-PCR (qRT-PCR) was performed on a 7500 Fast Real-Time PCR system (Applied Biosystems) using Fast SYBR Green master mix (Applied Biosystems). All annealing steps were carried out at 60 °C. Relative mRNA expression of target genes was calculated with the comparative CT method. All target genes were normalized to GAPDH in multiplexed reactions performed in triplicate. Differences in CT values ($\Delta$CT = CT gene of interest—CT GAPDH in experimental samples) were calculated for each target mRNA by subtracting the mean value of GAPDH (relative expression = ${2}^{-{\Delta} CT}$)^21,38^. Information on primer sets (Eurofins MWG Operon, Huntsville, AL, USA) used in this study is listed in Table 2S.

### Statistical analysis

Statistical analyses were performed using Excel and Prism 5 software (GraphPad Software, La Jolla, CA, USA). Flow cytometric data demonstrated a normal distribution and equal variation; hence only parametric tests were required. The exclusion criterion was set to be 1.5 S.E.M. from the mean. No data met this criterion, and thus all data points were included in the analysis. Statistical analysis for flow cytometry data was performed with one-way ANOVA followed by Tukey’s multiple comparison, and statistical analysis for qPCR was performed with Kruskal Wallis test followed by uncorrected Dunn’s multiple comparison test (both with 95% confidence intervals). Data were presented with the Mean ± S.E.M. A p-value less than 0.05 denoted statistical significance. The number of independent samples used for flow cytometry analysis is as follows: normal (n = 12), mild PE (n = 5), severe PE (n = 6) for MNC, and normal (n = 12), mild PE (n = 5), severe PE (n = 5) for CD31 cells. The number of independent samples used for qRT-PCR analysis was as follows: normal (n = 10), mild PE (n = 5), and severe PE (n = 4).

## Supplementary Information

Supplementary Information
