# Supplementary material for: Reduced angiovasculogenic and increased inflammatory profiles of cord blood cells in severe but not mild preeclampsia
Source: Sci Rep. 2021 Feb 11;11:3630. doi: 10.1038/s41598-021-83146-8 (PMC7878804; doi:10.1038/s41598-021-83146-8)
Supplement: Supplementary file 1 — Supplementary Information [file 41598_2021_83146_MOESM1_ESM.pdf]

Reduced Angiovasculogenic and Increased Inflammatory Profiles of Cord Blood Cells in Severe but not Mild Preeclampsia

Affiliations:

Seonggeon Cho BS[1], Young-Doug Sohn PhD[2], Sangsung Kim MS[4], Augustine Rajakumar PhD[3], Martina L. Badell MD[3], Neil Sidell PhD[3], and Young-sup Yoon MD, Ph.D[1,2,4]

[1]Coulter Department of Biomedical Engineering, Emory University and Georgia Institute of Technology, Atlanta, GA 30322, USA

[2]Department of Medicine, Division of Cardiology, Emory University, Atlanta GA.

[3]Department of Gynecology and Obstetrics, Emory University, Atlanta GA.

[4]Severance Biomedical Science Institute, Yonsei University College of Medicine, Seoul, South Korea.

First two authors (Cho, S and Sohn, Y-D) contributed equally to this manuscript.

*Correspondence to Young-sup Yoon, Division of Cardiology, Department of Medicine, Emory University School of Medicine, 101 Woodruff Circle, Woodruff Memorial Building (WMB) 3309, Atlanta, GA 30322. Email: yyoon5@emory.edu

Total word count of the manuscript: 4608

Supplemental Table

Table 1S. Surface Antigen Acronym and Function

Acronym	Description
CD3	T lymphocyte marker
CD11b	Integrin alpha M (ITGAM)<br>Leukocyte marker
CD14	Monocyte marker
CD19	B lymphocyte marker
CD31	Platelet endothelial cell adhesion molecule (PECAM1)
CD34	Hematopoietic stem/progenitor cell marker
KDR	Kinase insert domain receptor<br>Vascular endothelial growth factor receptor 2 (VEGFR2)<br>Fetal Liver Kinase 1 (FLK1)

Table 2S. List of Primers

Gene	Forward	Reverse
GAPDH (Glyceraldehyde-3-phosphate dehydrogenase)	F1: GGTGGTCTCCTCTGACTTCAACA<br>F2: GAGTCAACGGATTTGGTCGT	R1: GTGGTCGTTGAGGGCAATG<br>R2: TTGATTTTGGAGGGATCTCG
VEGFA (Vascular endothelial growth factor A)	CCCTGATGAGATCGAGTACATCTT	AACGCTCCAGGACTTATACCG
FGF2 (Fibroblast growth factor 2)	AGCGACCCTCACATCAAGCTA	CCAGGTAACGGTTAGCACACACT
ANGPT1 (Angiopoietin 1)	CAGAAAACAGTGGGAGAAGATATAACC	TGCCATCGTGTTCTGGAAGA
HGF (Hepatocyte growth factor)	CAATAGTCAATTTAGACCATCCCGTAAT	CGTGTTGGAATCCCATTTACAA
IGF1 (Insulin like growth factor 1)	CCATGTCCTCCTCGCATCTC	CGTGGCAGAGCTGGTGAAG
PGF (Placental growth factor)	CTGACGTTCTCTCAGCACGTTC	CTCCTTTCCGGCTTCATCTTC
PDGFB (Platelet derived growth factor subunit B)	CATTCCCGAGGAGCTTTATGAG	TCCAACTCGGCCCCATCT
CXCL12 (C-X-C motif chemokine ligand 12)	GAAGCGAAAAAATCAGTGAATAAACC	TGGAACCTGAAACCCTGCTG
IL1 (Interleukin 1)	GCGACGGTCACCTTCATC	AGGCACGTGAGCCTCTCTTT
IL6 (Interleukin 6)	GCTGCAGGCACAGAACCA	GCTGCGCAGAATGAGATGAG
IL8 (Interleukin 8)	GCTGCGCAGAATGAGATGAG	CTTGGCAAAACTGCACCTTCA
IL10 (Interleukin 10)	GCCGTGGAGCAGGTGAAG	TGGCTTTGTAGATGCCTTTCTCT
TNFA (Tumor necrosis factor α)	GGAGAAGGGTGACCGACTCA	CAGACTCGGCAAAGTCGAGATA
TGFB (Transforming growth factor beta)	TCCAACTCGGCCCCATCT	R1: ACCCGTTGATGTCCACTTGC<br>R2: TTGATGTCCACTTGCAGTGTGTTAT
